# Some Observations on Ulceration of the Kidneys; with Cases

**Published:** 1828-09

**Authors:** John C. Warren


Art. II.—*Some observations on Ulceration of the Kidneys, with cases*—By John C. Warren, M. D.

From the Boston Medical and Surgical Journal, No. 30.

The disease of ulceration of the kidneys is of not unfrequent occurrence. There is an obscurity in the symptoms of this distressing complaint, which causes it to be mistaken for diseases of the bladder, or of the urethra: and to the great distress and even destruction of the patient, the treatment is directed to the urethra or bladder instead of the real seat of the disease.

It is not in my power to dissipate the obscurity which involves the symptoms of this affection. But I have met with some cases in which the phenomena were observed with considerable care; and their causes afterwards verified by examination of the dead body. The publication of these will serve to call the attention of others to the subject; and lead to the discrimination of this obstinate complaint.

I will first state the case, and then add such remarks as they may suggest.

The first of these cases I visited in consultation with my friend, Dr. J. P. Spooner, to whom I am indebted for the history of the disease and dissection.

Case 1. Mr. ————, æt. 29, in Feb., 1823, complained of passing bloody urine. This occurred in greater or less quantity every day—generally more in the evening than in the morning. Sometimes the urine was slightly tinged; at other times a few drops of blood followed the evacuation; at others again the whole quantity was deeply tinged, so that it had to him the appearance of pure blood. It was increased after drinking of vinous and spirituous liquors,—after indulgence at the table,—after long sitting, as he was in the habit of doing with his feet raised above the level of the chair,—after bodily or mental fatigue. Occasionally after long sitting, or after having passed a large quantity of urine, he felt a pain in the region of the kidneys. With the exception of some symptoms of dyspepsia, the bloody urine and an occasional pain in the small of his back, were all of his complaints. He was directed a cathartic of pil. aloes, and colocynth and calomel,—a spare diet,—the constant use of a decoction of uva ursi,—and tincture of sulphuric acid. For a short time his symptoms seemed to abate. As the spring opened, he took a journey to the south, which was of benefit to his general health, but was of no service to his particular difficulty. Through the summer he complained but little, but in the autumn he became worse. The urine was more uniformly bloody, he often passed large clots of blood, and began to appear emaciated.

The sulphate of copper and the acetate of lead were successively resorted to, but as they both disagreed with the stomach and bowels, their use was discontinued. In December two issues were made, one over each kidney. The effect of these issues was a sudden diminution in the quantity of urine and also in the quantity of blood; but a new symptom now appeared, viz., a pain following the discharge of urine. An apparent improvement in his condition continued for several months, with the exception of the pain just alluded to, which became more and more troublesome.

March 15th, 1824. My notes say he has had no discharge of blood for a fortnight, excepting a slight tinge this afternoon. Within the last week he has had a pain attack him in the right knee, which passed to the calf and thence to the knee of the other leg. His bowels are still liable to derangement from slight causes. The issues continue to discharge, although they are smaller than they have been. April 1st. As the painful affection left the knee the discharge of blood returned. The pain of passing urine is now extreme, and is often accompanied with tenesmus to such a degree that it sometimes produces involuntary discharge of the fæces; followed by shortness of breath. Bowels very irregular; countenance pale and emaciated. By the advice of some of his physicians he sailed to day for Richmond.

June 1st. Returned worse; dysuria increased; very much emaciated; constant dry cough; difficulty in respiration. Gave him squill pills, applied leeches over the publis, and on going to bed gave him an injection of flaxseed tea and laudanum.

This treatment afforded entire relief to his pulmonary complaints, and very much mitigated his other sufferings. The injection was continued nightly for a long time with much benefit. But as the summer advanced, his complaints became worse. Frequent vomitings, with severe paroxysms of pain, seized him, his legs became œdematous, and he was constantly troubled with a dribbling of urine, for which he was obliged to wear oiled silk bags made for the purpose. In September he made a short visit to Nahant. After his return, his situation was truly distressing. He would be seized several times in the course of an hour with excruciating pains, which seemed almost insupportable; his legs were painfully distended; and he rejected with distressing retching almost every thing he took. Nothing could be done for his relief. The injections of laudanum and flaxseed had lost most of their good effects, and there was this objection to them that they seemed to increase the irritibility of the stomach. At this time, whilst I was assisting him into bed, he was seized with one of his paroxysms. I immediately pressed upon the small of the back with all my strength, at the same time passing my hands up and down. This gave him instantaneous and entire relief. I continued with him through the night, and resorted to the same method whenever he had warning of an approaching paroxysm, and uniformly with relief.

From the time of his returning, (20th of Sept.) from Nahant, he was no longer able to attend to his business.

By rest from the active duties of life, and by resorting to the method just stated, his distress was much mitigated; but he gradually declined until he died, on the 17th October, 1824, being twenty months from the time of his first complaining.

After death an examination was made by Drs. Ware, Doane, and Wells. The right kidney was found very much enlarged, being twice the common size, and of an unusually light color; its consistence not much altered. The left kidney not much altered. The ureters were much distended. The bladder was very much diseased, being in a scirrhous state, in some parts an inch or more thick; its cavity of course much diminished. The state of the bladder accounted for the distressing paroxysms of pain which he suffered, and which resembled the severe pains of a woman in labor, more than any thing to which I could compare them. And the appearance of the bladder reminded me of what we may suppose that of the uterus to be soon after parturition. The mucous membrane of the stomach and intestines was also much inflamed. The distention of the ureters can be accounted for from the state of the bladder, which presented a mechanical obstruction to the passage of the urine. Whether the primary disease occurred in the bladder or kidneys, it seems impossible to determine. But from the order of the symptoms I have been inclined to refer it to the kidneys.

Case 2. This case I saw in consultation with Dr. William J. Walker, of Charlestown, who has had the goodness to favor me with a history of the case and to send me the diseased kidney. He has added two other cases of remarkable disease in this same organ.

Mr. L. Lincoln, a married man of excellent character, correct habits, forty years of age, by trade a sailmaker, and who had never been affected with any venereal complaints, applied to me, three years since, on account of a serious disease of the urinary organs. He informed me that this disease commenced about eight years before that time, and could not be ascribed to any palpable cause. At first he had frequent calls to pass urine, attended with a heat and itching about the orifice of the urethra and glans penis. This difficulty at first appeared to yield to the use of nitrous ether and laudanum, combined with mucilages. This relief was, however, only momentary, for the complaint returned in paroxysms, and gradually became more severe, until it destroyed the comfort, health, and finally the life of its victim. At the commencement of my attendance I noted the following symptoms. He was somewhat wasted in flesh; his countenance was anxious; his pulse and skin natural, except when suffering from a parozysm of pain. At this time he would sweat freely, yet his pulse would be but little altered from its healthy state. He was unable to retain his urine more than one or two hours, and never voided more than four to six ounces at a time. The urine was always clear, never deposited a sediment, never contained either blood, mucus or pus, and always appeared of a healthy straw color. He now had a constant pain at the end of the yard, and a sense of weight about the rectum, which frequently induced him to go to stool without effect. This symptom was not dependent upon piles, for on examination per anum, I detected no disease of the anus, rectum, or prostrate gland. His urine frequently stopped suddenly during its excretion. His paroxysms were preceded and attended by a diminished flow of urine, and his relief always attended by a more copious flow of this fluid. A sound introduced into the bladder came in contact with no stone. Yet when its point was directed to a certain part of the bladder, it produced a sensation of touching some substance harder than the coats of the bladder, but without the shock which is felt when the sound comes in contact with a stone. The introduction of the sound gave more pain than is usual with others, but its use, and even its free use, was not followed by protracted pain, bleeding, or any sensible change in the urine. From the difficulty of the dianosis, I was induced to use the sound and to vary its form more freely than I should have done had I known the state of the bladder as found on dissection. During his paroxysms of suffering he constantly complained of the most acute pain in the right inguinal region. This spot he said he could cover with the point of his finger. His gait was peculiar. He walked with his legs wide apart, swinging forward as it were first one side of the body and then the other, without flexing the limbs, so as not to jar the system. While walking, he usually had the right hand in his trowsers, holding the penis. His body was constantly inclined to the right side, and although in the prime of life he had the stoop forward, and every way the motions, of a man in the decrepitude of age. As the constitution of my patient was naturally good, his digestive organs in perfect state, except when interrupted by the most agonising pain; as he had no febrile symptoms, and as all his sufferings had reference to the urinary apparatus only, he received no treatment, save only such as might probably obviate or mitigate the sufferings which he endured.

At the time of his greatest suffering he was three times bled by leeches. They were applied in considerable numbers to the perineum and the skin over the external abdominal ring. After each application he complained of more pain than ordinary, and said the bleeding hurt him. Of the truth of his suffering more for the leeching I am by no means certain; I am however, that it did him no good. Saline cathartics were of no service, if we except soda and Rochelle powders, when they did not prove too operative. Pyrola umbellata, uva ursi, gum Arabic decoctions of mucilaginous seeds, and mucilaginous drinks, generally gave some, yet but little relief. Nitrous ether, nitrous ether and oil of juniper, and bals. copavia, appeared sometimes of service, and sometimes worse than useless. Tonic medicines were aiways prejudicial. Fomentations, enemas, and all topical applications, failed of giving relief. Opium, belladonna, and conicum maculatum, did no other good than to relieve pain for the time. As to diet, stimulating food did harm. Tender succulent vagetables, farinaceous articles and ripe fruits, proved most friendly. He expressed great relief from the use of asparagus, berries, milk, &c. Whenever his suffering intermitted for a time, he would get a good appetite and increase in flesh; but a return of pain would soon reduce him to his former state.

Finally, worn down by pain, and despondent, he yielded himself to quackery, swallowed the infallible potions, and gave full credit to the last medicine used during a paroxysm, until a new period of pain convinced him of his mistake. A colliquative diarrhœa came on, which no medicine could restrain, and he died.

On opening the body I found no part diseased except the urinary organs. By removing the superjacent parts, the kidney appeared about four or five times its natural size; and the ureter, through its whole course as large as a man's thumb, was indurated, thickened, and apparently shortened. It was as it were stretched from one viscus to another, and might account for the constant inclination of the body to the right side. On removing the kidney and ureter from their natural situation, I found their cavities not materially enlarged. The increase of size arose from an increased thickness of the parenchymatious substance of the kidney and the coats of the ureter. The cavities of the kidney and ureter, were in a state of supporation. At this I was not a little surprised, as I have before stated that I had never detected in the urine either blood, pus or mucus. I at first thought that the ureter might be impervious, but by using the blowpipe, air passed readily into the bladder and to the kidneys. Here then we had an extensive suppurating cavity communicating with the bladder, and yet until within a few days of the patient's death no matter could be found in the urine. I say until within a few days of his death; for after my examination I learned of his wife that for the three or four last days of his life his urine looked as if a little milk had been mixed with it. Before that time I can answer that it did not exist, as I was in the habit of inspecting it frequently, and had enjoined it upon my patient to save for my own eye any thing deviating from a perfectly natural appearance in this excretion. Here sir let me ask, when did this suppuration commence! Within a few days of the patient's death? or at a much earlier period? Is it possible that so extensive a suppuration could have existed in these parts, and no matter be detected in the urine? and yet can it be possible that suppuration had not longer existed? considering the extent of disease, and the almost unequivocal marks of protracted ulceration and suppuration exhibited by the preparation now in your possession. On turning my attention to the bladder itself, I found its coats remarkably thin and weak, so much so that in endeavoring to separate it from the circumjacent cellular membrane, my fingers passed through into its cavity. In other cases of contraction of the size of the bladder, I believe the coats of this organ are thickened and strong; at least it has been so with all those cases which have come to my knowledge. The mucus membrane was changed from its natural appearance to a smooth substance, covered by minute granular shining points, which felt to the finger almost as hard as cartilage. The indulated part struck by the sound proved to be the end of the ureter where it enters the bladder. The sensation communicated to the fingers by touching this part of the bladder with the sound, the fact that the pain was principally in that place, and the difficulty of the diagnosis, led me to the belief that there might be a stone either impacted in the ureter or encysted in this part of the bladder. On viewing the smallness of the bladder and the tenuity of its coats, and reflecting on the freedom with which I had used the sound in this case, I could but be surprised that the point of this instrument had not done irreparable mischief.

Case 3. In the year 1815 I was requested by my preceptor and friend, (the late Governor Brooks) to examine a child of Mr. Calvin Turner, who had died of an immense tumor in the abdomen. The whole body, previous to any dissection, weighed twenty-seven pounds. Having laid open the abdomen, I found the tumor was one of the kidneys, which, when removed from its connexions, weighed fourteen pounds, being one pound more than the residue of the body. On opening this kidney, I found its upper extremity had degenerated into a firm white substance, almost cartilaginous, but becoming less white and less hard as it approached the middle part, where it had the color and natural appearance of the parenchymatous substance of the kidney in a natural state. The whole structure of this tumor resembled very nearly a natural kidney, allowance being made for its immense size. From its tubuli I pressed out urine having all the sensible properties of that fluid. Urine was also found in the pelvis of the kidney, and in the ureter. What the symptoms were which attended this case I cannot say, as I never saw the patient until I was called upon to make the dissection. I learned from Governor Brooks, however, that this was the third case of a similar disease which had occurred in his extensive practice, and that so little did the symptoms point to the precise nature of the disease, that in one case the spleen was supposed the part diseased, and in the other no satisfactory diagnosis could be formed till after dissection.

Case 4. About eleven years since I was requested to visit a Mr. Bailey, living in Tewksbury. The messenger who called on me, (himself a medical man,) wished me to go prepared to perform an operation upon a large tumor situated upon the patient's left side, and which was supposed to be a suppuration of the spleen. On arriving at my patient's house, I found a number of medical men who had convened to consult on the case, and to give assistance in any operation which might be deemed expedient. We found, by examining the side, a large tumor, which had the appearance of pointing in two different places. The one was near the margin of the false ribs, about four inches from the ensiform cartilage, and the other about four inches from the first in a line toward the posterior spinous process of the ileum. The skin over these points appeared reddish, and the parts thinner than at any other places. No one doubted that he felt fluctuation at these points, and even that fluctuation could be felt in the one by tapping on the other. The operation had been proposed to the patient by highly respectable gentlemen who had visited him previous to myself. All present thought it necessary. The patient and his wife ardently wished it done, expecting that a discharge of matter would give great relief. As the operation devolved upon me, and as I was at that time young in the profession, I went alone into my patient's room, that I might examine the case a little more carefully. This second thought proved fortunate for me, for by engaging the man in conversation, by altering his position in bed, by having his legs raised, while I made my investigations, and by similar manœuvres, I convinced myself that what appeared to be fluctuation might be only the slipping of a tumor under the parietes of the abdomen. Suspicion being roused, I pursued my inquiries until I was fully convinced that the safety of the surgeon as well as of the patient required forbearance. Perhaps the countenance of my patient, and his extreme emaciation, assisted me in the diagnosis. The countenance was that of a man laboring under incurable visceral disease; an appearance which I will not undertake to describe, but which the attentive observing practitioner will readily know, after having once seen it. I had now got myself into trouble, for the patient and his wife insisted on having the operation done, saying he could only die, and that this operation constituted his only hope. In addition to this, some of my medical friends could not give up their preconceived opinion. They quoted my words just given, that there was fluctuation in the tumor. Finally, with much difficulty, I persuaded my patient and friends that it might be better to wait two or three weeks and to have the opening made at that time, if he should still wish it, and if it should then appear feasible. Under these circumstances I left my patient, satisfied I had established a reputation for timidity, and equally satisfied that I had found in my composition so much of that better part of valor called discretion.

In a sdort time this man died. His body was examined by my much esteemed friend, Dr. Rufus Wyman. The tumor proved to be the kidney; was greatly enlarged; had formed no adhesions with the peritoneum opposite where it had pointed; was so soft in its texture that those present thought it contained a fluid until it was removed from the body and opened. This opening proved it to be of a soft spongy nature, and was thought by Dr. Wyman to be of the nature of fungus hæmatodes. This however was not all, for the colon, in a distended state, passed directly over the two projecting points, in such a manner as to have been infallibly opened by any incision into the tumor.

As this case had been attended by highly respectable practitioners, and as the urinary secretions and excretions had been so well performed as not to point out the parts diseased, may we not consider the following corollaries as established, viz., that great and even fatal disease may exist in the urinary apparatus, and yet not be discoverable by any known symptoms, and that a disease or irritation existing in one part of said apparatus, may produce symptoms of disease in any other part of the same.

Case 5. Charles E. Fessenden applied to me in the autumn of 1827. He had an inclination to pass water very frequently, with blood occasionally and a small quantity of mucous. The stream was rather small. I passed a bougie, and found an obstruction in the further part of the urethra. On two more applications this disappeared. He was not, however, relieved; nor did the medicines I afterwards gave, produce any mitigation of his symptoms. Satisfied that I should never be able to discover the nature of his complaint by the observations I could make in his occasional visits to me, I advised the young gentleman to go to the Hospital. The history of his case was there recorded as follows. Although long, I have not thought it best to omit any part of the history of the symptoms or treatment; as useful results may perhaps be drawn from them,—if not now, at some future time.

C. E. Fessenden, æt. 19, Boston. Entered the Hospital Dec. 3d, 1827. Constitution naturally good, but subject to frequent headache, and indigestion. Four months ago, received a severe strain in his back, from lifting a heavy weight. A fortnight after this, he began to feel a severe pain across the small of the back. After about a fortnight, this subsided, and was succeeded by a burning pain, about an inch from the extremity of the penis, coming on after micturition. Urine became darker colored than usual, depositing a mucous sediment. For the last three months has occasionally passed blood with his urine. Urine passed occasionally eighty times in the twenty-four hours, preventing his sleeping by the frequent necessity of rising.

The pain in the back recurred about a fortnight before he entered the Hospital. Subject to costiveness.

Dec. 4. Warm bath every other day.

℞ Potass. sup. carb. ʒij.

Aquæ, ℥vj.

Syrup. tolutan. ℥ iss.

Ol. junip. ℥ ss. M.

Cap. ℥ ss. ter in die.

May have meat; but no butter or fat.

6th. Sound passed, but no stone discovered in the bladder. Passed about a gill of blood.

℞ Sol. sulph magnes. ℥ iv.
Appl. hirud. vj. perineo, altern. diebus.

9th. Sound again passed, but no stone discovered.

Omitt. medicamenta.
Sum. pulv. uv. ursi, ℈ ij. ter in die.
Cap. pil. hyd. submur. et opii. nocte.

10th. Costive.

Sum. sol. mag. sulph. ℥ iv.

12th. Less pain after passing urine, and less frequent calls.—Pains across loins much diminished since entrance. Has passed but a few ounces of blood since the 5th.

Sum. sol. mag. sulph. ℥ ij. altern. diebus, mane.

17th. Improved every way. Warm bath only twice a week.

27th. General improvement. Mouth slightly affected. Less tickling about penis since entrance. Has a new sensation of burning just within the urethra and in the bladder before passing urine, relieved after evacuation. Passes urine four or five times daily; quantity as usual. Mucous sediment continues, but no blood for a considerable time.

Omitt. sol. mag. sulph.

Jan. 1. Doing well. Burning in penis and bladder, before passing urine, continues. No pain in any part. No blood.

Appl. hirud. bis in hebdomada.

14th. Mouth continues slightly sore.

18th.

Sum. pil. submur. et opii. bis in die.

25th. Calls to pass urine of late more frequent. For several days severe but momentary pain, in end of penis after passing water. Has to-day severe pain across small of back, chiefly before and after passing urine.

Omitt. uv. ursi et. pil.
Sum. balsam copaib. gtts. xxx. in aq. ter in die.
Csntinue bath.

28th. Has taken cold and looks less well. Pain in back, and pain after passing water continue. For several days has constantly passed blood with urine. Since balsam, quantity of urine diminished; more frequent calls. On the 26th, passed urine sixty times.

Mucous sediment increased. Also burning in penis and bladder before passing urine. Ardor urinæ lately; none now.

Appl. empl. canth. iij. ad vj. dig. dorso.

30th. Better; has not taken the balsam very regularly. May walk out.

Appl. part. vesicat. unguent. junip.
Omitt. balsamum.

Feb. 2. Sharp aching pain near the extremity of the penis, commencing yesterday about 1, P. M. and lasting till 8 or 9. Very little burning pain in penis, and very little pain in back since blister was applied. None since yesterday morning. Less disturbed in night by necessity of passing urine. Urine in larger quantities. Sleep better than usual. Bowels regular.

3d. Pain in back recurred yesterday afternoon after a dinner of salt fish, and lasted about seven hours. Had a very uncomfortable night. Passed urine about four times. Severe pain after passing it; slight burning pain before; Urine scalding.

4th. Yesterday and last night more comfortable. Slight aching pain in penis for about an hour. Pain in back as on the day before.

5th.

℞. Pulv. uv. ursi, ʒj. ter in die.

6th. Less pain in back; recurred about three times in the day, lasting about fifteen minutes at a time. Burning pain for about half an hour after micturition. Urine diminished in quantity. Passed about eight times in the day; four times in the night. Pain before micturition less severe. Urine scalding. Bowels free.

8th. No recurrence of pain in back. Burning pain in penis as usual. Pain in extremity of penis before micturition. No pain last night after micturition. Slept well. Urine as usual. Pain for about two hours this morning; relieved by drinking slippery elm tea. This morning much increased by motion; this has not been the case before. Bowels free.

11th. Has continued to gain since the 6th. No recurrence of pain in the back. Pains in penis slight. Appetite good. Health good. Has lost in weight during the last fortnight, but never before.

12th. Passes urine eight times a day; four times in the night. Passes no blood. Mucous sediment rather less.

℞. Tinct. mur. ferri et ammon. gtts. xv. ter in die.

Sum. inf. lini. semin. ℥ ij. quarta quaque hora.

℞. Tinct. capsic. ℥ iv.

Tinct. canth. ℥ j. M.

Utet. pro lotione dorso.

Cont. baln. tepid. et uva ursi.

13th. Continues to improve. Has not now the sensation of weariness which he formerly experienced at night. Strength increases. Has gained flesh.

14th. Continues to amend. Allowed, at request, to go out this evening.

16th. Feels much better than at any time since his entrance.—Urine passed about twenty times a day.

18th. Has been worse since the morning of the 16th. Pains increased. Urine passed more frequently, in smaller quantity, and with more sediment.

19th.

Omitt. omnia preter lotiones et balneum.

℞. sol. sulph. mag. ℥ iv.

20th. Much better this morning. Night very good. Twelve dejections from medicine, copious. Sharp burning pain yesterday after micturition, for about half an hour at a time. This is much increased, and is often brought on by motion. Severe itching pain this morning, lasting about half an hour at a time.

21st.

℞. Hyd. submur.
Pil. scillæ, aa gr. xij. M. Ft. pil. No. xij.
Cap. unam, mane et vespere. Cont cetera.

22d. Worse since yesterday's visit. Urine, according to report, somewhat tinged with blood; otherwise as before.

23d.

Omitt. pil. mane.

26th. Has been much worse since the 22d, to-day especially. Pains severe, recurring constantly upon exercise. Feeling of great weariness. Little appetite. Uneasiness, though not exactly pain, in the back. Pain in left groin, over a circular space of the size of a dollar, previous to passing urine. Bowels generally irregular when not under the influence of medicine. At present rather costive.

Omitt. hirud. applic.
Sum. pil. hyd. submur. mane et vespere.

27th. Last night and this morning much better. Takes daily a quart of the decoction of slippery elm.

28th. Much better.

March 1. Feverish.

℞. Spirit. æth. nitros. gtts. xl.

Quarta quaque hora. Cont. pil. hyd. submur.

℞. Acid. sulphur. ʒij.

Ol. olivæ, ʒij. M.

Appl. dorso pro linimento, mane et vespere.

3d. Pains have rather increased in consequence of his taking cold. Pain in the back for about half an hour yesterday. Passes urine about thirteen times in the night. Great weakness, especially at night.

4th. Urine passed twenty times in the night; in sufficient quantity, rather light colored, depositing some reddish flocculi. Vomited this morning after breakfast.

℞. Ant. tart. gr. x.

Aquæ ℥ iij. M.

Cap. ℥ ss. semi horis, pro emetico.

6th. Pains increased on account of his eating salt, by which his symptoms are always exacerbated.

℞. Pulv. uv. ursi, ℈ ij. ter in die.

Cont. cetera.

7th. Much better.

8th. Tongue furred, and other symptoms of fever.

℞. Hyd. submur.

Pulv. jalapæ, aa gr. viij. M.

Cap. statim.—Omitt. medicament. solita.—Liquid diet.

10th. Was quite weak and in bed nearly all day on the 8th and yesterday. After dinner yesterday, was seized with vomiting, and paroxysms of violent pain ensued. Appearance under the paroxysms, and symptoms, were as follows:

Countenance very much distressed and pale; writhing in bed; scalding in urethra, as from a hot iron passed into it; excruciating pain in the end of the penis, and in the left testicle, descending from the symphysis pubis; itching in the penis within the perineum; pulse smaller than usual, not much accelerated; feet very cold; on examination, slight retraction of testicle, but otherwise the appearance natural. Pains came on immediately after passing urine. This attack continued about two hours, when he became relieved. A second paroxysm, similar to the former, commenced at 6, P. M.

During the first paroxysm he was directed by the house physician to take slippery elm tea, and continue this as long as the pain lasted. Fomentation of hops applied about the pubes, and kept hot. While this is preparing, foment with hot water. Enema of tepid flaxseed tea, half a pint, and laudanum, seventy-five drops.

Second paroxysm, opiate enema, tr. camph. opiat. gtts. xl.; spirit. æth. nit. gtts. xxx. M. In the evening, hop fomentation, with a strong decoction of poppies. At 9, spirit. æth. nit. gtts. xlv.; repeated at half past 11. At 2. A. M. tinc. opii. gtts. xxv.

This attack left him very feeble, and he continued in bed for two or three days, but had no severe pain, and gradually mended.

15th. Return to usual diet.

Cont. pil. mane et vespere.

17th. Countenance continues pallid. Passes urine about fifteen times in the night, with some clots of blood. Sediment somewhat increased.

19th. Passed a very comfortable night. Bladder again sounded. Passing the catheter into the bladder produced exquisite pain. Felt in the bladder, as he said, like a red hot iron.

Cap. pil. unam vespere.

Appl. empl. canth. dig. iv. abdomini.

24th. Complains of an itching about the anus, which he says he has experienced from the commencement of the disease. Has lost fourteen pounds of flesh within a fortnight.

Diet,—for dinner, fish, oysters, broiled chicken; for breakfast, eggs, toasted bread, crackers, shells, cocoa or arrowroot.

Sum. pulv. uv. ursi, gr. xx. ter in die.
℞. Hyd. submur.
Camphoræ,
Opii. aa gr. vj. M. Ft. pil. No. vj.
Cujus, cap. unam vespere.

27th. Urine within the last twenty-four hours, eighty ounces.—Has gained strength.

Sum. uv. ursi, ℈j. ter in die.

28th. Severe pain in the lower part of the abdomen.

Appl. empl. canth. digit. iv. abdomini.

31st. Slight relief to abdominal pain from blister. Nights un easy.

Sum. vespere, ext. hyoscyami, gr. vj.

April 4. No effect from hyoscyamus.

Omitt. pil. hyos. et sum. pil. camph. et opii. gr. vj. nocte.

5th. Slept well. Took two pills.

6th. Recurrence of paroxysm similar to those of March 10th, and nearly as severe.

℞. Spirit. æth. nitros. ℥ ss.

Tr. oppi, ℈ij. M.

Cujus cap. ℈j. quaque hora, ad allev. doloris.

7th.

Habet. baln. tepid. nocte.

Sum. sol. mag. sulph. ℥ iv. crass mane.

11th. Advised to lay aside the use of medicines, to go into the country as soon as he is able, have two issues on the back, and use no hard water. Have all his food boiled in soft water.

Upon the 12th, the weather being fine, and he thinking his strength sufficient to leave the Hospital, he was discharged. Some cause prevented his going that day, however, and on the 13th the paroxysms recurred a third time, with the additional symptom of pain in the back. Had an injection, from which two dejections, one copious.

14th.

Sum. inf. senn. comp. ℥ ij. et post horas sex, enema si opus sit. Si dolor redeat, appl. foment. tanacet. et absinth. parti affectæ.

℞. Spirit. æth. nitros. ℥ ss.
Ol. junip. gtts. xxx. M.
Cujus cap. gtts. xxx. tertia quaque hora.
Omitt. pil. hodie.

15th.

Si dolores. red. appl. empl. canth. dig. iv. ad vj.

16th. This morning in a state of stupor. Answers questions with difficulty, and incorrectly. Loss of memory. This state continued till eleven or twelve o'clock, when he recovered, and soon after left the Hospital.

In about three days after leaving the Hospital, he died.

Examination April 24th, the day after death.

Slight adhesion of the omentum to the muscles on the left side. Left kidney considerably enlarged, with decided appearances of inflammation on exterior surface, covered with whitish spots, softer than natural. In the interior, appearances of inflammation much greater. Various ulcerations were discovered in the pelvis of the kidney, six or eight in number, and situated in the infundibula, and mamillary eminences. Ureter on this side enlarged to above four times the natural size, parietes very much thickened, and passage larger than natural. Right kidney larger than natural, with appearances of inflammation and spots of ulceration, but less than on the left side. Ureter enlarged. Bladder very small, its parietes much thickened; mucous coat of an ash color, with streaks of vermillion, and interspersed with numerous black dots. Prostate gland small, with no appearances of disease. Two singular appendages to the spleen, of the same substance with that organ, of the appearance of small lobes.

### REMARKS.

On the last case I would remark, that when the patient first presented himself to me, I had a strong expectation of finding a stone in the bladder; his symptoms, as described to me, being very similar to those of the latter affection, with the exception of the sudden obstruction of the urine while passing, which he had not. When I attempted to pass an instrument, an obstruction presented itself in the membranous part of the urethra. This was removed in a few days without any mitigation of the symptoms, and the examination of the bladder was made, but no stone discovered. I then examined the prostate gland. This was found quite natural in feel, though smaller than usual. Having disposed of the suspicion of these complaints, I next directed my attention to the state of the urinary bladder. On examining externally, in different directions and situations, especially over the pubes, no tenderness could be discovered. There was however occasional pain above the pubes, a little to the left side; and the passage of an instrument into the bladder demonstrated an excessive tenderness in the internal coat of this organ. On repeated observations, I was not satisfied that an inflammation of the mucous membrane of the bladder in a patient of this age, could assume the obstinate character manifested by this complaint: and reflecting that the disease began with a violent pain in the back, I became satisfied that the original seat of it was the kidneys; and this inference was drawn perhaps as much from negative, as positive signs. The opinion was then stated to those who visited the patient with me.

On examination of the body after death, I proceeded in the first instance to the left kidney, which was as I had believed in a diseased state; though the extent of disease was much less than might be expected from such severe and fatal symptoms. The mucous coat of the bladder was clearly in a state of chronic inflammation; but its condition would by no means justify the suspicion of its being the principal seat of disease. We conclude that the disease of this young man began in the kidneys, and was the effect of an injury operating on a delicate and irritable habit.

When we consider the general aspect of the cases related, we must be struck at first view with the intensity of the symptoms. In this respect the affection does not correspond with the other derangements of these organs, so far as they have been discriminated. A sudden enlargement of the prostate gland, which sometimes takes place in middle aged persons, and might, in opposition to the chronic disease of old age, be called sub-acute inflammation of the prostate, is accompanied by most violent and distressing symptoms; but they are easily discriminated to be the symptoms of affection of this gland, and moreover, an examination will show the gland in a very uncommonly enlarged state. The existence of a stone in the bladder is also accompanied by very severe symptoms; but they may be distinguished from those of the disease of which we are speaking, by being less constant.

This character of intensity will therefore lead to a suspicion of affection of the kidneys.—From other diseases occurring in the urinary organs it may be distinguished also.

First. From structure in the urethra, by the want of pus in the urine in stricture; by the narrowness and obstinacy of the contraction; and by the great length of time usually required for the formation of the structure. It must be noticed, however, that in disease of the kidneys there is often a sympathetic contraction of the urethra, which misleads the surgeon to the opinion that the complaint is stricture. But in this case the contraction will be found to vary extremely, sometimes to disappear and afterwards show itself anew in its original state. There are frequently mistakes committed in examinations for structure. Many a patient has been subjected to the action of a caustic, because a plaster bougie would not pass; when if a metallic cathetre had been employed, the largest instrument would have freely entered the bladder. Before deciding on the nature of one of these cases, every kind of instrument should be employed, and at different periods.

Second. Disease of the kidneys may be distinguished from enlargement of the prostate gland by examination. To form a correct judgment in this case, the surgeon must be much in the habit of making examinations of this gland in its healthy and morbid state.

Third. From chronic inflammation of the bladder it will be extremely difficult to distinguish this affection of the kidneys. There is however, I suspect, reason to doubt whether chronic inflammation of the bladder does frequently occur as a primary disease. Perhaps it may be for want of observation, or from want of a sufficient number of dissections; but I do not recollect a case of chronic inflammation of the mucous membrane, as a primary and uncomplicated disease. In the cases of ulceration of the kidney, however, this coat has always exhibited signs of severe inflammation after death, and sometimes during life. A question may arise whether the inflammation of the mucous coat were a primary or secondary affection in the diseases of the kidney. I cannot doubt for a moment that the ulceration of the kidney is the original disease, and that the mucous coat is affected secondarily. The case of simple chronic inflammation of the mucous coat of the bladder, will be attended with symptoms less violent and less obstinate than the disease of which we are speaking. It might be thought that the pain in the back would serve to distinguish the affection of the kidneys from that of the bladder. But this pain is not so prominent a symptom as might be expected. In the case of Fessenden it scarcely existed except in the beginning of the disease.

Fourth. This disease will be distinguished from stone in the bladder by examination; and also by the sudden stop of the flow of urine, by the stone falling on the orifice of the urethra.

*Symptoms of the disease.* The disease sometimes begins with a pain in the back, but not uniformly. This is followed by an increased desire to pass water, which is done very often and in small quantities. As the disease advances this disposition becomes excessive, and much greater than what is known in other affections of these parts, frequently more than sixty times in twenty-four hours. The urine is slightly tinged with blood, and mixed with mucous. This mucous is afterwards purulent; or rather there is a mixture of blood and foul pus in variable proportions, sometimes the one and sometimes the other predominating. The pain is often excessive. Sometimes it is in the course of the urethra, accompanied by a violent burning; sometimes in the extremity of the penis as in the stone, and frequently in the lower part of the abdomen, in the middle or one side. The passage of an instrument into the bladder produces great pain. The pulse is accelerated, and tongue furred, though in a changeable manner. Sometimes there are regular paroxysms of fever, preceded by chills. The constitution becomes greatly disabled. Exhalations take place in the cellular membrane. The nervous system is disturbed. Under these distressing symptoms the patient survives for a considerable time.

*Treatment.* Whether this disease is ever curable I know not. I have a patient who has been ill three years, and for some time lay in a hopeless condition. He is now able to walk, ride, eat and drink, as usual; yet I cannot say he is well.—In the beginning of the complaint it would be proper to take blood from the arm, afterwards to apply leeches frequently to the loins and the perineum. The warm bath is useful. Mucilaginous injections into the rectum, with the addition of tincture of opium, when the pain is severe. Deep issues should be made with caustic, on each side of the spine; or on one side only if the disease seems to be confined to one kidney. Mucilaginous drinks, especially decoction of flaxseed, drank very copiously, give more direct ease than any other remedies taken into the stomach; for in general, medicines passed through the stomach appear to have no great influence on the complaint. All stimulants are injurious. The balsams therefore do harm Injections into the bladder, however mild, are pernicious. The patient's regimen should be of the mildest kind; he should avoid the use of spring water, and employ only pure water, either distilled or rain water. Finally, the symptoms must be mitigated by the constant use of opium. Hyoscyamus, stramonium and hemlock, do not appear to be substitutes for this medicine.

